# Hyperoxia in critically ill patients with sepsis and septic shock: a systematic review

**DOI:** 10.1186/s44158-023-00096-5

**Published:** 2023-05-03

**Authors:** Francesca Romana Catalanotto, Mariachiara Ippolito, Alice Mirasola, Giulia Catalisano, Marta Milazzo, Antonino Giarratano, Andrea Cortegiani

**Affiliations:** 1 Department of Surgical, Oncological and Oral Science (Di.Chir.On.S), University of Palermo, 90127 Palermo, Italy; 2 Department of Anaesthesia, Intensive Care and Emergency, Policlinico Paolo Giaccone, Via del Vespro 129, 90127 Palermo, Italy; 3 Azienda Ospedaliera Ospedali Riuniti Villa Sofia Cervello, Palermo, Italy

**Keywords:** Sepsis, Septic shock, Oxygen, ICU, Hyperoxia

## Abstract

**Background:**

In septic patients, hyperoxia may help with its bactericidal effects, but it may cause systemic impairments. The role of hyperoxia and the appropriate oxygen target in these patients is unknown. The aim of this systematic review was to summarize the available literature.

**Methods:**

We conducted a systematic search screening PubMed and Cochrane Library. Studies on adult patients with sepsis or septic shock and admitted to ICU addressing the topic of hyperoxia were included and described.

**Results:**

We included 12 studies, for a total of 15.782 included patients. Five studies were randomized controlled trials (RCTs) or analyses from RCTs, three were prospective observational studies, and four were retrospective observational studies. The definition of hyperoxia was heterogeneous across the included studies. Mortality was the most frequent outcome: six studies showed an increased rate or risk of mortality with hyperoxia, three found no differences, and one a protective effect of hyperoxia. At the critical appraisal assessment stage, no major methodological flaws were detected, except for a single-center, pilot study, with a lack of adjustment for confounders and imbalance between the groups.

**Conclusion:**

The optimum range of oxygen level able to minimize risks and provide benefits in patients with sepsis or septic shock seems still unknown. Clinical equipoise between hyperoxia and normoxia is uncertain as conflicting evidence exists. Further studies should aim at identifying the best range of oxygenation and its optimal duration, investigating how effects of different levels of oxygen may vary according to identified pathogens, source of infection, and prescribed antibiotics in critically ill patients with sepsis and septic shock.

**Supplementary Information:**

The online version contains supplementary material available at 10.1186/s44158-023-00096-5.

## Background

Sepsis and septic shock are leading causes of mortality and morbidity in patients admitted to the intensive care unit (ICU). In the pathophysiology of septic shock, an imbalance occurs between oxygen supply and oxygen consumption [1]. Therefore, many ICU patients with sepsis require vasopressors, invasive ventilation, and the provision of supplemental oxygen. However, the appropriate regimen of oxygen administration is unknown [2]. The Surviving Sepsis Campaign Guidelines [3] stated that there is insufficient evidence to make a recommendation on the use of conservative oxygen targets in adults with sepsis-induced hypoxemic respiratory failure, thus not providing any threshold for arterial oxygen partial pressure (PaO_2_) or arterial oxygen saturation (SaO_2_). Although oxygen therapy is essential in most critically ill patients, they may be exposed to high level of oxygen and develop a hyperoxia status, potentially determining harm. The effects of a high PaO_2_ are controversial: on the one hand, oxygen has bactericidal properties, but on the other hand, hyperoxemia seems also able to cause systemic complications. Indeed, an excess of oxygen availability may result in the production of reactive oxygen (ROS) [4, 5] alteration of mitochondrial respiration, activation of apoptosis pathway, atelectasis [6], and vasoconstriction [7]. Moreover, in vitro studies showed that exposure to different levels of oxygen may modify the sensitivity of bacteria to antibiotics [8]. Therefore, oxygen levels may influence the outcome of septic patients through several mechanisms.

In literature, many studies have been published in recent years, evaluating the effects of hyperoxemia in the setting of critical care, some showing that hyperoxia may increase mortality, especially in settings like traumatic brain injury, and others the return of spontaneous circulation after cardiac arrest [9–11]. However, the role of hyperoxia in patients with sepsis or septic shock remains unclear. Therefore, we aimed at summarizing the available evidence on the role of hyperoxia in critically ill patients with sepsis or septic shock and the association between hyperoxia and mortality and other clinical outcomes (e.g., hemodynamics, renal function, etc.), as investigated by the available literature.

### Main text

For the purpose of this review, we performed a systematic search in PubMed and The Cochrane Library database, lastly updated on 17 April 2023. We included the following search key terms: “sepsis” or “septic shock,” “hyperoxia” and “critical care” and related synonyms, alternatives, and plural. The full search strategy is available in Supplementary Material 1. The reference list of relevant articles was also screened (i.e., the snowballing method). The systematic review was conducted as per PRISMA guidelines [12].

Studies were independently screened from titles and abstract by two authors (F.R.C., A.M.) to identify all the relevant records and screened from full text against inclusion and exclusion pre-defined criteria by the same authors. Differences were resolved by consensus with a third author (M. I.). Eligibility criteria included studies assessing the effects of hyperoxia in adults (≥ 18 years) admitted to the critical care for sepsis or septic shock. We included studies independently of definition of hyperoxia. Studies including less than 10 patients, case reports, abstracts, review articles, and articles in languages different than English were excluded. We also excluded studies conducted on pediatric patients and animal studies. No studies were excluded for their outcomes. Authors, publications, date of publication, hyperoxia definition, and primary and secondary outcomes were extracted from each original article and were tabulated. The included studies were then assessed using JBI’s Critical Appraisal Checklists (https://jbi.global/critical-appraisal-tools) [13–15], according to their designs.

A total of 725 records were retrieved. After the screening of the records and removal of duplicates, 33 records were evaluated from full text, of whom 21 were excluded and 12 studies were included, for a total of 15,782 included patients. All patients received supplemental oxygen, and the majority were mechanically ventilated. At the critical appraisal assessment stage, no major methodological flaws were detected, except for a single-center, hypothesis-generating pilot observational study, with lack of adjustment for confounders and unclear balance of patients’ characteristics between the groups [16]. No studies were excluded at this stage.

**Fig. 1:**
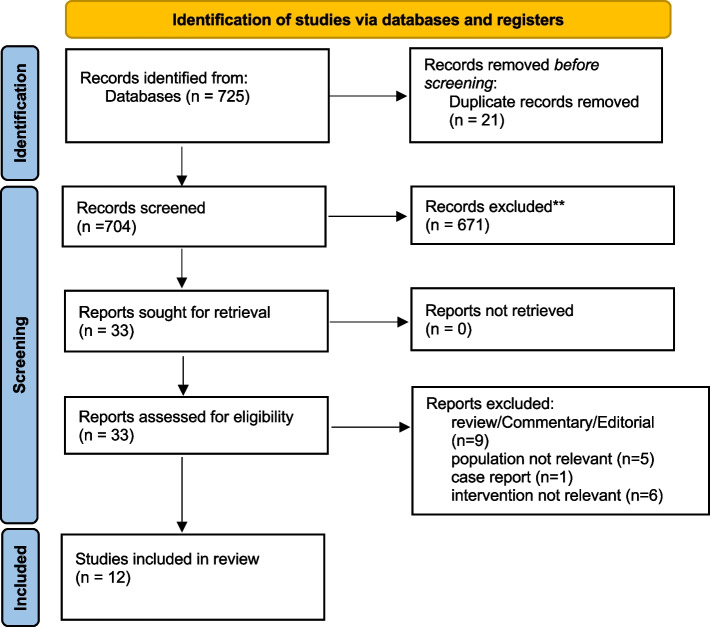
PRISMA 2020 flow diagram

The inclusion/exclusion process is presented with details as a PRISMA flow diagram, as shown in Fig. 1. The included studies comprised 1 randomized clinical trial (RCT), 4 secondary analyses from RCTs, 3 prospective observational studies, and 4 retrospective observational studies. The comparison group, present in 10 studies, was normoxia, and the most frequently investigated outcomes were mortality, intensive care unit-acquired weakness, atelectasis formation, length of stay in the ICU, incidence of renal-replacement therapy and acute kidney Injury (AKI), days to suspension of vasopressor or inotropic agents, and the percentage of resolution of primary and secondary infections, mechanical ventilation duration, vascular effects, oxidative stress, and the incidence of sepsis-associated encephalopathy (SAE). The main characteristics of the included studies are described in Table 1. The PRISMA checklist is available as Supplementary Material 2.

**Table 1:** Characteristics of the included studies

**Author (year)**	**Design of the study**	**Population**	**Setting**	**Definition hyperoxia**	**Comparison group**	**Oxygen therapy**
Asfar [17] 2017	Multicenter RCT	434 patients with septic shock (aged > 18 years)	ICU	FiO_2_ at 1.0 for 24h after inclusion	FiO_2_ set to target SapO_2_ of 88–95% (normoxia)	Mechanical ventilation
Carr [18] 2020	Secondary analysis *of* RCT	27 septic patients subcohort, from 125 patients under mechanical ventilation	ICU	Usual oxygen (no specific measures limited FiO_2_ or SpO_2_)	Conservative oxygen therapy (FiO_2_ was reduced as much as possible down to a minimum of 0.21 maintaining 90% < SpO_2_ < 97%)	Mechanical ventilation
Catalisano [19] 2023	Secondary analysis of RCT	1632 septic patients who survived the first 48 h since randomization	ICU	PaO_2_ > 100 mmHg	PaO_2_ ≤ 100 mmHg	79% of patients received mechanical ventilation
Demiselle [20] 2018	Secondary analysis *of* RCT	397 patients with septic shock	ICU	FiO_2_ 1.0	FiO_2_ set to target SapO_2_ of 88–95% (normoxia)	Mechanical ventilation
Jouffroy [21] 2019	Single-center retrospective observational study	49 patients with septic shock	ICU	PaO_2_ > 150 mm Hg	PaO_2_ < 100 mmHg, 100 < PaO_2_ < 150 mmHg	Pre-hospital mechanical ventilation
Kota Nishimoto [22] 2021	Single-center retrospective study	213 mechanically ventilated septic patients	ICU	Conventional oxygenation target SpO_2_ ≥ 96%	Conservative targets with permissive hypoxia (SpO_2_: 88–92% or PaO_2_: 60 mmHg) and hyperoxia avoidance (reduced oxygenation for PaO_2_ > 110 mmHg)	Mechanical ventilation
Martín-Fernández [23] 2022	Secondary analysis of a prospective observational study	454 patients who underwent major surgery admitted into a single ICU	ICU	PaO_2_ > 100 mmHg	PaO_2_ ≤ 100 mmHg	Invasive mechanical ventilation
Young [24] 2019	Secondary analysis of RCT	251 patients with sepsis	ICU	Usual oxygen (no specific measures limited FiO_2_ or SpO_2_)	Conservative oxygen therapy (FiO_2_ was reduced as much as possible down to a minimum of 0.21 maintaining 90% < SpO_2_ < 97%)	Mechanical ventilation
Yun Li [25] 2022	Retrospective observational study	11740 septic patients	ICU	PaO_2_ > 339 mmHg, PaO_2_/FiO_2_ > 619	NA	47% of patients received mechanical ventilation
Popoff [26] 2021	Retrospective observational study	488 mechanically ventilated patients with septic shock	ICU	PaO_2_ > 120 mmHg	70 < PaO_2_ < 120 mmHg	Mechanical ventilation
Rossi [27] 2007	Single-center prospective observational study	14 patients with severe sepsis or septic shock requiring mechanically controlled ventilation	ICU	FiO_2_ 1.0	NA	Invasive mechanical ventilation
Stolmeijer [16] 2014	Single-center prospective observational study	83 patients admitted with two or more SIRS criteria and a suspicion of an infection	Emergency department	PaO_2_ > 13.5kPa (approx. > 101 mmHg)	PaO_2_ < 9.5 kPa (approx. < 71 mmHg)	VentiMask (FiO_2_ 0.4) or nonrebreathing mask (FiO_2_ 0.6–0.8)

#### Oxygen therapy in sepsis and septic shock

**Fig. 2:**
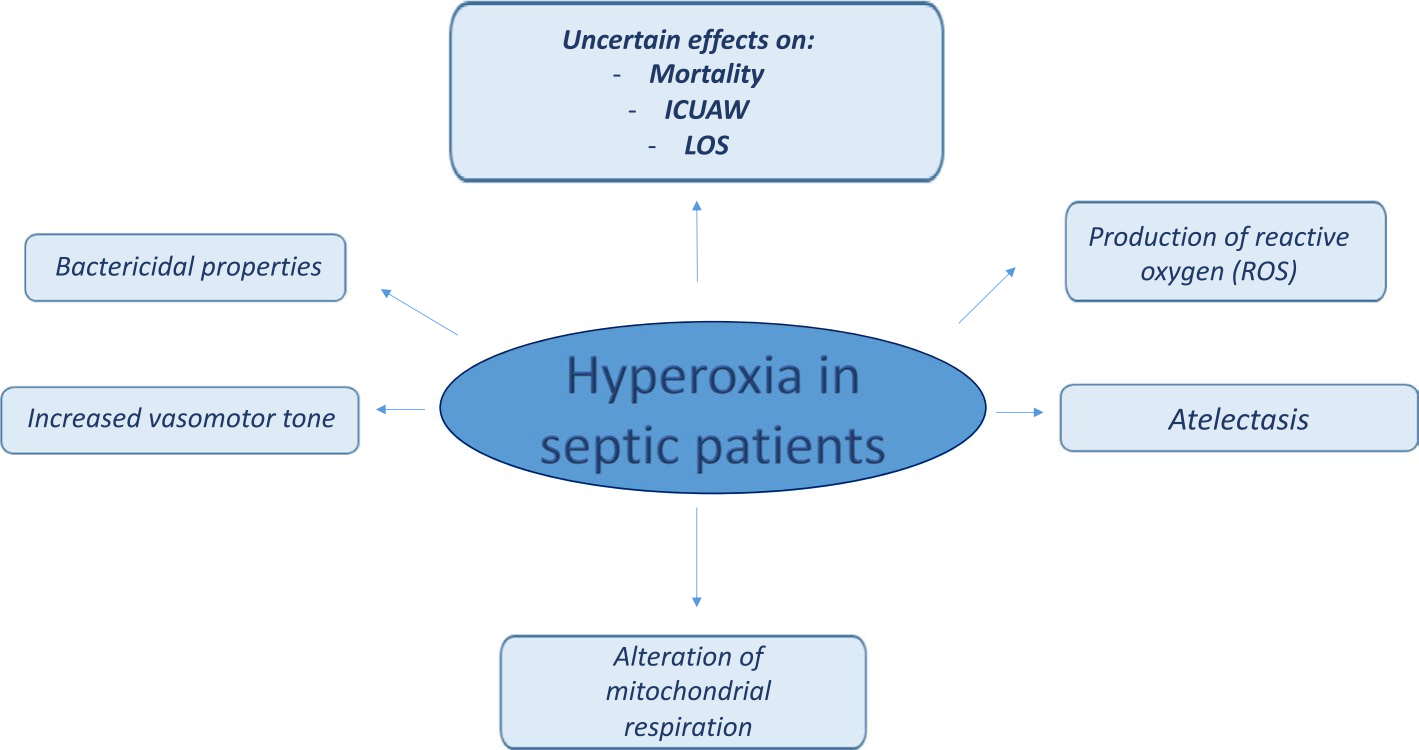
Proposed physiologic effects and clinical impact of hyperoxia in patients with sepsis. The figure summarizes the physiologic effects of hyperoxia and its role on clinical outcomes in patients with sepsis or septic shock. ICUAW, intensive care unit-acquired weakness; LOS, length of stay; ROS, reactive oxygen species

Sepsis is a medical emergency; therefore, early diagnosis and appropriate management improve outcome [28–30]. Treatment is based on early and appropriate antimicrobial therapy, source control, fluid resuscitation, and eventually (e.g., septic shock) the use of vasoactive medications and mechanical ventilation [31]. Patients often receive oxygen supplementation [32]. However, the Surviving Sepsis Campaign Guidelines [28] do not provide indication on targets for the partial pressure of oxygen in arterial blood or arterial oxygen saturation. The physiologic effects of hyperoxia and its role on clinical outcomes have been graphically summarized in Fig. 2.

#### Definitions of hyperoxia

A high incidence of hyperoxia has been described in septic patients, reaching an average of 92.8% in a prospective study [16] conducted on 83 septic patients, despite being included according to an old definition. The effects of hyperoxia in patients with sepsis or septic shock have received increasing interest over the last three decades [33].

Hyperoxia was differently defined across the included studies. Some studies considered as belonging to the “hyperoxia group” the patients receiving a fixed FiO_2_ of 1.0 [17, 20, 27]. Some trials used a threshold of PaO_2_ > 100 mmHg [16, 23], PaO_2_ > 120 mmHg [26], PaO_2_ > 150 mmHg [21], or SpO_2_ > 96% [22] to define hyperoxia status. The absence of a uniform definition may be one of the main issues on the topic, both in the clinical setting and in the research field.

#### Pathophysiology

Oxidative cellular damage has been widely studied in medical research and has been associated with an impaired mitochondrial activity and the production of reactive oxygen species (ROS) [4, 5]. Breathing with excess oxygen may increase the formation of ROS, such as hydroxyl radical (OH•) and peroxynitrite (ONOO−), able to interact with lipids, proteins, and nucleic acids [34], thus determining a direct oxidative stress [35] and an indirect damage through radical-mediated mechanisms, inducing cells to undergo necrosis or apoptosis. Moreover, neutrophils can use oxygen to form superoxide and other reactive oxygen species that, despite beneficial in the killing of microorganisms, may become risky in the context of a dysregulated host response such as sepsis. From a hemodynamic perspective, hyperoxia induces systemic vasoconstriction through the ROS [36] production and the low bioavailability of NO [37]. ROS production has also been considered among the possible mechanism of ICU­acquired weakness [38]. Absorption atelectasis [39] are important pulmonary effects, along with pulmonary cellular damage [40] and decreased mucus clearance [40]. Indeed, when using high FiO_2_, alveolar nitrogen, that is an inert gas, is gradually replaced by oxygen and washed out, thus determining alveolar collapse once that oxygen is absorbed into the blood.

Overall, the pathophysiological effects of hyperoxia in sepsis are controversial. On the one hand, supplemental oxygen can be life-saving in such patients, and even hyperoxia may be useful due to its bactericidal effects, but on the other hand, a use of high FiO_2_ may cause systemic impairments.

Moreover, the devices adopted to deliver oxygen may also have non-oxygen-related effects that may be considered as confounders of the net effect of oxygen per se. Indeed, septic patients have an increased respiratory drive and usually high spontaneous efforts [41–43], and it has been shown that the use of HFNC may reduce respiratory drive in such patients, compared with low-flow oxygen therapy, and contribute to maintain a state of normoxia [44, 45] by determining washout of dead space, compensating excessive carbon dioxide production due to a hypermetabolic state, and provide expiratory positive pressure [46, 47], overall reducing the work of breathing [44].

#### Mortality in sepsis/septic shock

Mortality was an assessed outcome in 10 of the included studies. Of these, 6 found a higher mortality rate or an increased risk of mortality among patients with sepsis/septic shock and hyperoxia [24, 25], 3 found no difference in mortality between the two groups [16, 22, 26], and 1 found a reduced risk of mortality [23] among patients with hyperoxia.

A secondary analysis of a prospective observational study [23], which included 454 postsurgical patients with sepsis or septic shock and need for invasive mechanical ventilation, showed that hyperoxia, defined as PaO_2_ > 100 mmHg during the first 48 h after major surgery, was associated with a lower risk of 90-day mortality (OR 0.61, 95% CI: 0.39–0.95, *p* = 0.029), compared to PaO_2_ < 100 mmHg, independently of age, presence of chronic renal failure, procalcitonin levels, or APACHE II score. Patients were first treated with empirical antibiotic therapy waiting for susceptibility testing to be completed, with subsequent targeted therapy selected according to the results. Specifically, linezolid or teicoplanin was used for methicillin-resistant *Staphylococcus aureus* and at least one of the following antibiotics for *Pseudomonas aeruginosa*: imipenem, cefepime, or piperacillin-tazobactam, in association with amikacin or ciprofloxacin.

Two retrospective cohort studies did not find any significant association between hyperoxia and ICU mortality in mechanically ventilated septic patients. In the first [26] one, hyperoxia was defined as PaO_2_ > 120 mmHg during the first 24 h of ICU stay, and the study included 488 patients with septic shock, defined according to SEPSIS-3 criteria. The second one [22] evaluated 83 patients treated with conventional oxygenation targets (SpO_2_ target of ≥ 96%) and 130 patients with permissive hypoxia (SpO_2_ target of 88–92% or PaO_2_ target of 60 mmHg; reduction of FiO_2_ if PaO_2_ > 110 mmHg). There was no statistically significant difference in ICU mortality (*p* = 0.18).

Stolmeijer et al. conducted a single-center prospective observational study [16] including a small sample size of 83 septic patients and found no significant differences between hyperoxia and normoxia groups in terms of in-hospital and 28-day mortality. However, the outcomes of this study must be considered in the context of limitations typical of the study design.

No association has been found between survival and hyperoxia in a recent post hoc analysis [19] of the ALBIOS RCT. The authors included 1632 septic patients who survived the first 48 h after randomization and stratified them into two groups based on their mean PaO_2_ levels during the first 48 h (PaO_2_ 0–48 h) with a cutoff of 100 mmHg (mean PaO_2_ 0–48 h > 100 mmHg: hyperoxemia group *n* = 971; PaO_2_ 0–48 h ≤ 100: normoxemia group *n* = 661). The data analysis did not show any significant difference between the two groups regarding mortality at 90 and 28 days. However, a subgroup analysis performed in the same study and including patients with lung as the primary site of infection (*n* = 663) showed a reduced risk of mortality at 90 days in patients with hyperoxemia.

Four of the included studies found an increase in mortality in the group of patients with hyperoxia. The multicentric RCT HYPERS2S [17] by Asfar et al. compared the effects of hyperoxia (FiO_2_ 1.0 for 24h after inclusion) with normoxia in 434 patients with septic shock who were on mechanical ventilation. The study was prematurely terminated due to a higher 28-day mortality in the group receiving hyperoxia. In this study, hyperoxia was associated with higher risk of mortality, although not statistically significant; 28-day mortality was recorded for 434 patients; 93 (43%) of 217 patients had died in the hyperoxia group versus 77 (35%) of 217 patients in the normoxia group (HR 1.27 (95% CI 0.94–1.72); *p* = 0.12).

A post hoc analysis [20] of the same study compared mortality rates in the 397 patients in whom lactate levels were available at baseline to compare a Sepsis-3 [48] shock subset (lactate > 2 mmol/L) of patients to those with vasopressor-dependent hypotension only (lactate ≤ 2 mmol/L). Hyperoxia treatment for 24 h compared to “normoxia” was associated with a higher mortality rate in patients with septic shock defined as per the Sepsis-3 definition (57.4% vs. 44.3%, *p* = 0.054). In patients with lactate ≤ 2 mmol/L, hyperoxia had no effect on mortality (*p* = 0.680).

Young et al. undertook a post hoc analysis [24] of the ICU-ROX trial, on the subcohort of 251 patients with sepsis. Indeed, the ICU-ROX trial had compared conservative oxygen therapy (FiO_2_ reduced as much as possible down to a minimum of 0.21, maintaining SpO_2_ < 97%), with usual oxygen therapy (no specific thresholds for FiO_2_ or SpO_2_) in 1000 mechanically ventilated patients admitted to ICU. In the secondary analysis, the conservative oxygen therapy group did not result in a statistically significant reduction of 90-day mortality (95% CI − 4.6 to 18.6% points; *p* = 0.24) compared with the usual oxygen group in septic patients. However, the authors discussed that the analysis was underpowered to detect the effect on 90-day mortality.

A single-center retrospective observational study [21], conducted on 49 septic patients subjected to assisted-mechanical ventilation before hospital admission, showed that hyperoxia, defined as PaO_2_ > 150 mmHg at ICU admission, was associated with mortality at day 28 in septic patients, using a propensity score analysis including SOFA score, pre-hospital duration, lactate, and pre-hospital fluid volume expansion (*p* = 0.02, OR [CI95] = 1.59 [1.20–2.10]) [21]. However, a strong limit of the study was the unknown duration of hyperoxemia, impossible to determine because the included patients had been treated by a mobile intensive care unit and subjected to invasive mechanical ventilation prior to hospital admission. Further studies are currently ongoing on the topic, also investigating mortality as a primary outcome, and would reasonably contribute to producing useful data on the topic (NCT04198077).

Finally, another observational cohort study [25] was conducted on a sample of 11740 septic patients undergoing oxygen therapy in the ICU or perioperative period, selected from the MIMIC IV and eICU databases. The authors observed a directly proportional correlation between oxygen therapy and the incidence of sepsis-associated encephalopathy (SAE). SAE refers to cognitive dysfunction attributable to a systemic inflammatory response in the absence of direct CNS infections (defined in this study as GCS < 15 and/or patients diagnosed with delirium). The authors observed higher mortality rates among septic patients who developed SAE compared to those who did not and higher PaO_2_ and PaO_2_/FiO_2_ values among non-survivors of patients who developed SAE. The observational nature of the study is certainly a limitation; however, they observed that the range of PaO_2_ (97–339) mmHg, PaO_2_/FiO_2_ (189–619), and SpO_2_ ≥ 93% reduced the incidence of SAE and may reduce the hospital mortality of SAE. Instead, hypoxia (SPO_2_ < 93%, PaO_2_ < 97 mmHg, and PaO_2_/FiO_2_ < 189) and hyperoxia (PaO_2_ > 339 mmHg and PaO_2_/FiO_2_ > 619) were associated with increased incidence of SAE. Thus, lower or higher oxygenation could induce SAE.

#### Other outcomes

Ten additional outcomes were evaluated among the included studies: intensive care unit-acquired weakness, atelectasis formation, length of stay in the ICU, incidence of renal-replacement therapy and acute kidney injury (AKI), days to suspension of vasopressor or inotropic agents, and the percentage of resolution of primary and secondary infections, mechanical ventilation duration, vascular effects, and oxidative stress.

In the HYPERS2S RCT, a higher number of patients with intensive care unit-acquired weakness (24 [11%] vs 13 [6%]; *p* = 0.06) and atelectasis (26 [12%] vs 13 [6%]; *p* = 0.04) within the first 3 days was found in the hyperoxia group [17] compared with the normoxia group. There were no significant differences in the secondary outcomes: length of stay in the ICU (*p* = 0.49) and requirements for renal replacement treatment (*p* = 0.74). In the secondary analysis of a prospective observational study written by Martín-Fernández et al., hyperoxemia (PaO_2_ > 100 mmHg) was associated with a lower length of ICU stay (5 [9] vs. 8 [13] days, *p* < 0.001) and reduced mechanical ventilation duration (1 [4] vs. 2 [8] days, *p* < 0.001).

In a single-center retrospective study [22], a reduced ICU stay (11.0 [IQR: 6.0–19.0] days vs. 9.0 [IQR: 4.0–15.0] days, *p* = 0.02) and in mechanical ventilation duration (11.0 [IQR: 6.0–19.0] days vs. 7.0 [IQR: 3.0–14.0] days, *p* = 0.01) were found between the conventional oxygenation target and conservative targets groups.

In the recent post hoc analysis of an RCT [19], AKI, the percentage of patients undergoing renal replacement therapy, the suspension times of vasopressor or inotropic agents, the resolution of the primary infection, and mortality in UTI were not significantly different between the study groups. Conversely, a reduction in mechanical ventilation time and intensive care stay was found in patients with normoxemia compared to the hyperoxemia group.

Rossi et al. [27] in a prospective study evaluated the vascular effects during mechanical ventilation in 14 septic patients. After a 20-min period of hyperoxic ventilation (FiO_2_ 1.0), two-dimensional images of the brachial artery cross-sectional area and brachial blood flow velocities were recorded using conventional ultrasonography and pulsed Doppler simultaneously with invasive arterial pressure measurements. They observed a reduction in brachial cross-sectional areas and an increase in MAP of about 7%, an increase in pulse pressure and in resistance index, and a decrease in distensibility coefficient and in cross-sectional, showing that vasomotor tone increases. Vasoconstriction as a response to hyperoxia seems to result in a paradoxical decrease in arterial oxygen delivery, due to an impaired arterial blood flow, at least for the upper limbs.

In a sub-study of the ICU-ROX RCT [18] on 27 septic patients, the correlation between hyperoxemia (SpO_2_ ≥ 97%) and increased oxidative stress was evaluated comparing levels of ascorbate (one of the most potent water-soluble antioxidants in human plasma) and protein carbonyls (a biomarker of protein oxidation). From the data analysis, it emerged that conservative oxygen therapy did not alter systemic markers of oxidative stress in critically ill ventilated patients with sepsis compared with standard oxygen therapy.

### Limitations

This review has limitations. The main limitation was represented by the heterogeneity of the hyperoxia definition adopted across the studies, limiting the chance to further summarize and analyze data. Furthermore, the majority of the included studies did not provide detailed data on the causative microorganisms, antibiotic administration, or hyperoxia duration, which are expected to contribute to mortality as confounding variables, and the effects of blood oxygen levels on sensitivity to antibiotics were not investigated. Lastly, the small sample size in many of the selected trials does not allow for generalizable results.

## Conclusions

Conflicting evidence emerges from the included studies, but data from RCTs issued safety concerns on the use of hyperoxia in patients with sepsis or septic shock and potential association with higher mortality. The heterogeneity of the definitions adopted for hyperoxia hampers the chance to further summarize the available data. The optimum range of oxygen level able to minimize risks and provide benefits seems still unknown. Clinical equipoise between the two conditions (i.e., hyperoxia and normoxia) is uncertain in this population of patients, thus limiting future research options. Future studies should aim at (i) identifying the best range of oxygenation and its optimal duration to maximize benefits and minimize harm and (ii) investigating how effects of different levels of oxygen may vary according to identified pathogens, source of infection, and prescribed antibiotics in critically ill patients with sepsis and septic shock.

## Supplementary Information

**Additional file 1:** **Supplementary Material 1.** Full search strategy.

**Additional file 2:** **Supplementary Material 2.** PRISMA checklist.

## Data Availability

The datasets used and/or analyzed during the current study are available from the corresponding author on reasonable request.

## Abbreviations

CNS: Central nervous system
FiO_2_: Fraction of inspired O_2_
HFNC: High-flow nasal cannula
ICU: Intensive care unit
MAP: Mean arterial pressure
PaO_2_: Partial pressure of oxygen
RCT: Randomized controlled trial
ROS: Reactive oxygen species
SAE: Sepsis-associated encephalopathy
SaO_2_: Saturation of oxygen
