# Supplementary material for: Hyperoxia in critically ill patients with sepsis and septic shock: a systematic review
Source: J Anesth Analg Crit Care. 2023 May 3;3:12. doi: 10.1186/s44158-023-00096-5 (PMC10245526; doi:10.1186/s44158-023-00096-5)
Supplement: Supplementary file 1 — Additional file 1: Supplementary Material 1. Full search strategy. [file 44158_2023_96_MOESM1_ESM.docx]

Supplementary Material to:

Hyperoxia in critically ill patients with sepsis and septic shock: a systematic review

Francesca Romana Catalanotto ^1^, Mariachiara Ippolito ^1,2^, Alice Mirasola^1,3^, Giulia Catalisano^1^, Marta Milazzo ^1^, Antonino Giarratano^1,2^ and Andrea Cortegiani^1,2^*

^1^ Department of Surgical, Oncological and Oral Science (Di.Chir.On.S) University of Palermo, 90127 Palermo, Italy;

^2^ Department of Anaesthesia, Intensive Care and Emergency, Policlinico Paolo Giaccone, 90127 Palermo, Italy

^3^ Azienda Ospedaliera Ospedali Riuniti Villa Sofia Cervello

*** Corresponding Author:** Prof. Andrea Cortegiani, MD. Department of Surgical, Oncological and Oral Science (Di.Chir.On.S.), University of Palermo, Italy. Department of Anaesthesia, Intensive Care and Emergency, Policlinico Paolo Giaccone, Palermo, Italy, Via del Vespro 129, 90127 Palermo, Italy. Email: andrea.cortegiani@unipa.it; Tel.:+390916552730

**PUBMED**

**Search strategy**

((sepsis) OR (septic shock) OR (toxic shock) OR (endotoxic shock)) AND ((hyperoxia) OR (hyperoxemia) OR (liberal oxygen therapy) OR (conservative oxygen therapy) OR (high blood oxygen levels) OR (oxygen toxicity)) AND ((intensive care) OR (critical care) OR (critically ill) OR (ICU))

("sepsis"[MeSH Terms] OR "sepsis"[All Fields] OR ("shock, septic"[MeSH Terms] OR ("shock"[All Fields] AND "septic"[All Fields]) OR "septic shock"[All Fields] OR ("septic"[All Fields] AND "shock"[All Fields])) OR ("shock, septic"[MeSH Terms] OR ("shock"[All Fields] AND "septic"[All Fields]) OR "septic shock"[All Fields] OR ("toxic"[All Fields] AND "shock"[All Fields]) OR "toxic shock"[All Fields]) OR ("shock, septic"[MeSH Terms] OR ("shock"[All Fields] AND "septic"[All Fields]) OR "septic shock"[All Fields] OR ("endotoxic"[All Fields] AND "shock"[All Fields]) OR "endotoxic shock"[All Fields])) AND ("hyperoxia"[MeSH Terms] OR "hyperoxia"[All Fields] OR "hyperoxemia"[All Fields] OR (("liberal"[All Fields] OR "liberalization"[All Fields] OR "liberalize"[All Fields] OR "liberalized"[All Fields] OR "liberalizing"[All Fields] OR "liberally"[All Fields] OR "liberals"[All Fields] OR "politics"[MeSH Terms] OR "politics"[All Fields] OR "liberalism"[All Fields]) AND ("oxygen inhalation therapy"[MeSH Terms] OR ("oxygen"[All Fields] AND "inhalation"[All Fields] AND "therapy"[All Fields]) OR "oxygen inhalation therapy"[All Fields] OR ("oxygen"[All Fields] AND "therapy"[All Fields]) OR "oxygen therapy"[All Fields])) OR (("conservancies"[All Fields] OR "conservancy"[All Fields] OR "conservancy s"[All Fields] OR "conservation"[All Fields] OR "conservational"[All Fields] OR "conservations"[All Fields] OR "conservative"[All Fields] OR "conservatively"[All Fields] OR "conservatives"[All Fields] OR "conserve"[All Fields] OR "conserved"[All Fields] OR "conserves"[All Fields] OR "conserving"[All Fields]) AND ("oxygen inhalation therapy"[MeSH Terms] OR ("oxygen"[All Fields] AND "inhalation"[All Fields] AND "therapy"[All Fields]) OR "oxygen inhalation therapy"[All Fields] OR ("oxygen"[All Fields] AND "therapy"[All Fields]) OR "oxygen therapy"[All Fields])) OR ("high"[All Fields] AND ("oxygen saturation"[MeSH Terms] OR ("oxygen"[All Fields] AND "saturation"[All Fields]) OR "oxygen saturation"[All Fields] OR ("blood"[All Fields] AND "oxygen"[All Fields] AND "levels"[All Fields]) OR "blood oxygen levels"[All Fields])) OR (("cell respiration"[MeSH Terms] OR ("cell"[All Fields] AND "respiration"[All Fields]) OR "cell respiration"[All Fields] OR "oxygenation"[All Fields] OR "oxygen"[MeSH Terms] OR "oxygen"[All Fields] OR "oxygen s"[All Fields] OR "oxygenate"[All Fields] OR "oxygenated"[All Fields] OR "oxygenates"[All Fields] OR "oxygenating"[All Fields] OR "oxygenations"[All Fields] OR "oxygenative"[All Fields] OR "oxygenator s"[All Fields] OR "oxygenators"[MeSH Terms] OR "oxygenators"[All Fields] OR "oxygenator"[All Fields] OR "oxygene"[All Fields] OR "oxygenic"[All Fields] OR "oxygenous"[All Fields] OR "oxygens"[All Fields]) AND ("toxic"[All Fields] OR "toxical"[All Fields] OR "toxically"[All Fields] OR "toxicant"[All Fields] OR "toxicant s"[All Fields] OR "toxicants"[All Fields] OR "toxicated"[All Fields] OR "toxication"[All Fields] OR "toxicities"[All Fields] OR "toxicity"[MeSH Subheading] OR "toxicity"[All Fields] OR "toxicity s"[All Fields] OR "toxics"[All Fields]))) AND ("critical care"[MeSH Terms] OR ("critical"[All Fields] AND "care"[All Fields]) OR "critical care"[All Fields] OR ("intensive"[All Fields] AND "care"[All Fields]) OR "intensive care"[All Fields] OR ("critical care"[MeSH Terms] OR ("critical"[All Fields] AND "care"[All Fields]) OR "critical care"[All Fields]) OR ("critical illness"[MeSH Terms] OR ("critical"[All Fields] AND "illness"[All Fields]) OR "critical illness"[All Fields] OR ("critically"[All Fields] AND "ill"[All Fields]) OR "critically ill"[All Fields]) OR ("intensive care units"[MeSH Terms] OR ("intensive"[All Fields] AND "care"[All Fields] AND "units"[All Fields]) OR "intensive care units"[All Fields] OR "icu"[All Fields]))

**Translations**

**sepsis:** "sepsis"[MeSH Terms] OR "sepsis"[All Fields]

**septic shock:** "shock, septic"[MeSH Terms] OR ("shock"[All Fields] AND "septic"[All Fields]) OR "septic shock"[All Fields] OR ("septic"[All Fields] AND "shock"[All Fields])

**toxic shock:** "shock, septic"[MeSH Terms] OR ("shock"[All Fields] AND "septic"[All Fields]) OR "septic shock"[All Fields] OR ("toxic"[All Fields] AND "shock"[All Fields]) OR "toxic shock"[All Fields]

**endotoxic shock:** "shock, septic"[MeSH Terms] OR ("shock"[All Fields] AND "septic"[All Fields]) OR "septic shock"[All Fields] OR ("endotoxic"[All Fields] AND "shock"[All Fields]) OR "endotoxic shock"[All Fields]

**hyperoxia:** "hyperoxia"[MeSH Terms] OR "hyperoxia"[All Fields]

**liberal:** "liberal"[All Fields] OR "liberalism's"[All Fields] OR "liberalization"[All Fields] OR "liberalize"[All Fields] OR "liberalized"[All Fields] OR "liberalizing"[All Fields] OR "liberally"[All Fields] OR "liberals"[All Fields] OR "politics"[MeSH Terms] OR "politics"[All Fields] OR "liberalism"[All Fields]

**oxygen therapy:** "oxygen inhalation therapy"[MeSH Terms] OR ("oxygen"[All Fields] AND "inhalation"[All Fields] AND "therapy"[All Fields]) OR "oxygen inhalation therapy"[All Fields] OR ("oxygen"[All Fields] AND "therapy"[All Fields]) OR "oxygen therapy"[All Fields]

**conservative:** "conservancies"[All Fields] OR "conservancy"[All Fields] OR "conservancy's"[All Fields] OR "conservation"[All Fields] OR "conservational"[All Fields] OR "conservations"[All Fields] OR "conservative"[All Fields] OR "conservatively"[All Fields] OR "conservatives"[All Fields] OR "conserve"[All Fields] OR "conserved"[All Fields] OR "conserves"[All Fields] OR "conserving"[All Fields]

**oxygen therapy:** "oxygen inhalation therapy"[MeSH Terms] OR ("oxygen"[All Fields] AND "inhalation"[All Fields] AND "therapy"[All Fields]) OR "oxygen inhalation therapy"[All Fields] OR ("oxygen"[All Fields] AND "therapy"[All Fields]) OR "oxygen therapy"[All Fields]

**blood oxygen levels:** "oxygen saturation"[MeSH Terms] OR ("oxygen"[All Fields] AND "saturation"[All Fields]) OR "oxygen saturation"[All Fields] OR ("blood"[All Fields] AND "oxygen"[All Fields] AND "levels"[All Fields]) OR "blood oxygen levels"[All Fields]

**oxygen:** "cell respiration"[MeSH Terms] OR ("cell"[All Fields] AND "respiration"[All Fields]) OR "cell respiration"[All Fields] OR "oxygenation"[All Fields] OR "oxygen"[MeSH Terms] OR "oxygen"[All Fields] OR "oxygen's"[All Fields] OR "oxygenate"[All Fields] OR "oxygenated"[All Fields] OR "oxygenates"[All Fields] OR "oxygenating"[All Fields] OR "oxygenations"[All Fields] OR "oxygenative"[All Fields] OR "oxygenator's"[All Fields] OR "oxygenators"[MeSH Terms] OR "oxygenators"[All Fields] OR "oxygenator"[All Fields] OR "oxygene"[All Fields] OR "oxygenic"[All Fields] OR "oxygenous"[All Fields] OR "oxygens"[All Fields]

**toxicity:** "toxic"[All Fields] OR "toxical"[All Fields] OR "toxically"[All Fields] OR "toxicant"[All Fields] OR "toxicant's"[All Fields] OR "toxicants"[All Fields] OR "toxicated"[All Fields] OR "toxication"[All Fields] OR "toxicities"[All Fields] OR "toxicity"[Subheading] OR "toxicity"[All Fields] OR "toxicity's"[All Fields] OR "toxics"[All Fields]

**intensive care:** "critical care"[MeSH Terms] OR ("critical"[All Fields] AND "care"[All Fields]) OR "critical care"[All Fields] OR ("intensive"[All Fields] AND "care"[All Fields]) OR "intensive care"[All Fields]

**critical care:** "critical care"[MeSH Terms] OR ("critical"[All Fields] AND "care"[All Fields]) OR "critical care"[All Fields]

**critically ill:** "critical illness"[MeSH Terms] OR ("critical"[All Fields] AND "illness"[All Fields]) OR "critical illness"[All Fields] OR ("critically"[All Fields] AND "ill"[All Fields]) OR "critically ill"[All Fields]

**ICU:** "intensive care units"[MeSH Terms] OR ("intensive"[All Fields] AND "care"[All Fields] AND "units"[All Fields]) OR "intensive care units"[All Fields] OR "icu"[All Fields]

**COCHRANE**

**Search strategy**

(sepsis) OR (septic shock) OR (toxic shock) OR (endotoxic shock) in All Text AND (hyperoxia) OR (hyperoxemia) OR (liberal oxygen therapy) OR (conservative oxygen therapy) OR (high blood oxygen levels) OR (oxygen toxicity) in All Text AND (intensive care) OR (critical care) OR (critically ill) OR (ICU) in All Text - in Cochrane Protocols, Trials, Special Collections (Word variations have been searched)
